# Camrelizumab for cancers in patients living with HIV: one-single center experience

**DOI:** 10.1186/s12981-023-00518-y

**Published:** 2023-04-16

**Authors:** Menghua Wu, Xin Zheng, Yu Zhang, Jian Song, Jimao Zhao

**Affiliations:** 1 Department of Urology, Capital Medical University, Beijing Youan Hospital, Beijing, China; 2 Department of Urology, Capital Medical University, Beijing Friendship Hospital, Beijing, China

**Keywords:** Immune checkpoint inhibitor, People living with HIV, Advanced cancers, Camrelizumab

## Abstract

**Objectives:**

The primary objective was to evaluate the safety of the anti-PD-1 antibody camrelizumab in people living with HIV (PLWH); the secondary objective was to evaluate tumor response.

**Methods:**

From May 8, 2018, to December 10, 2021, twenty-four patients with HIV and advanced cancer as well as a CD4^+^ T-cell count greater than or equal to 100 cells/µL were treated with camrelizumab in daily practice. We describe the demographic characteristics, safety, and clinical course of these 24 PLWH with cancer treated with camrelizumab. Safety was assessed using the current Common Terminology Criteria for Adverse Events (CTCAE). The tumor response was assessed according to the Response Evaluation Criteria in Solid Tumors, version 1.1 (RECIST 1.1).

**Results:**

The median number of cycles was 8 (4–26). Only two grade 3 adverse reactions were reported (no toxic deaths or immune-related deaths). Among the 24 patients, 2 (8%) complete responses and 6 (25%) partial responses were observed. 7 patients (29%) were at stable tumor status and others progressed.

**Conclusions:**

Data from the present study strongly support the use of camrelizumab (monoclonal antibodies targeting the PD-1 pathway) in this population, as it appears to be a feasible approach with no deleterious effects on PLWH and tolerability and acceptable efficacy. In addition, these findings further support the inclusion of PLWH with cancer in clinical trials evaluating the safety and efficacy of ICIs on cancer.

## Highlights

Camrelizumab had an acceptable safety profile in 24 participants with HIV, a CD4+ T-cell count of greater than 100 cells/μL, and advanced cancer. This study increases confidence in immunotherapy for HIV.

## Background

With the advent of highly active antiretroviral therapy in recent years, people living with HIV (PLWH) have become less likely to die of AIDS-related illnesses. However, non-AIDS-related malignancies have been identified as the leading cause of death in PLWH. Compared with the general population, PLWH are at a higher risk of cancer [1–3]. The treatment regimens and outcomes in select patients with several cancers are like those in the general population [4, 5]. Currently, there is no standard therapy for PLWH with metastatic or locally advanced cancers, those with previous therapy failure due to disease progression or relapse, or those ineligibles to receive standard therapy [1].

Immune checkpoint inhibitors (ICIs) have emerged as a powerful new tool in cancer treatment. Immunotherapy has received considerable critical attention; however, current research and guidelines lack information on immunotherapy in PLWH advanced cancers [6]. Immunotherapy in PLWH may have beneficial treatment outcomes. As the indications for ICIs expand to various cancers, PLWH have been excluded from clinical immunotherapy studies. However, several case reports have reported on the immunotherapy treatment of PLWH with different types of advanced cancer. Although treatment with ICIs appears to be generally well tolerated in PLWH in these studies [6–9]. Lack of knowledge about using cancer therapies in PLWH, health care disparities, and HIV-associated immunosuppression may affect outcomes [10]. Camrelizumab, a highly-affinity, fully humanized, selective IgG4 monoclonal antibody against PD-1, has shown activity across a wide range of solid tumors [11, 12]. Yet, anti-PD-1 safety data are needed to guide the treatment of patients with HIV and to inform HIV-related eligibility criteria for future immune therapy.

The adverse event (AE) profile of checkpoint inhibitors targeting PD-1、PD-L1 has been tested in the general population with cancer and the immune-related AEs related to anti-PD-1 therapy occur in fewer than 30% of patients with cancer. The most common AEs are with skin, musculoskeletal, gastrointestinal, and endocrine, being generally mild to moderate [13]. Safety and efficacy data are lacking for ICIs in PLWH, because these patients have been systematically excluded from clinical trials. So, we determined to evaluate the safety and tumor response of camrelizumab in PLWH with advanced cancers.

## Methods

### Study design

This retrospective observational study included patients diagnosed with locally advanced or metastatic cancers who received treatment in our hospital from 8th May 2018 and 10th July 2021. Included cases were followed up by telephone until June 2022. Patients ’clinical data were taken from electronic health records in the hospital information system. The clinical and laboratory data were retrospectively retrieved by telephone and hospital medical case records. This study was subject to approval by the Ethics Review Committee of our hospital.

### Patient selection

Inclusion criteria were the following: (1) with controlled HIV viral load; (2) the baseline CD4 + T-cell counts > 100 cells/µL; (3) with no central nervous system metastases, (4) with complete data, including general laboratory and radiological data, related oncology treatment data ; (5) with an Eastern Cooperative Oncology Group (ECOG) performance score of ≤ 1;(6) not AIDS-defining cancers.

### Data collecting

A clinical physician collected data available on the case report form, including characteristics, baseline CD4^+^ T-cells count, HIV viral load before immunotherapy, and the number of cycles received. Camrelizumab (200 mg) wea administered intravenously every 2 weeks until death or intolerability. Treatment continued until disease progression, unacceptable toxicity, patient withdrawal, or investigator decision. The tumor response outcome was assessed by the radiographic data according to Response Evaluation Criteria in Solid Tumors, version 1.1 (RECIST 1.1). The adverse events were collected by the follow-up according to the Common Terminology Criteria for Adverse Events, version 4.03.

Changes in CD4 + T-cell count from baseline to the time of last treatment cycle were evaluated by Wilcoxon signed rank test. The cut-off time for follow-up was until May 2022, after the completing the last cycle or death. SPSS (version 26) were used for statistical analysis.

### Endpoints

The primary objective was to assess the safety and tolerability of camrelizumab in patients with HIV on ART with locally advanced or metastatic cancer. Immune-related AEs of grade 2 or higher were considered immune-related events of clinical interest. The secondary objective was to produce preliminary insights into clinical benefits. Tumor response was assessed by clinical trial group criteria, and clinical benefit was defined as complete response (CR) and partial response (PR).

## Results

### Patient characteristics

**Fig. 1:**
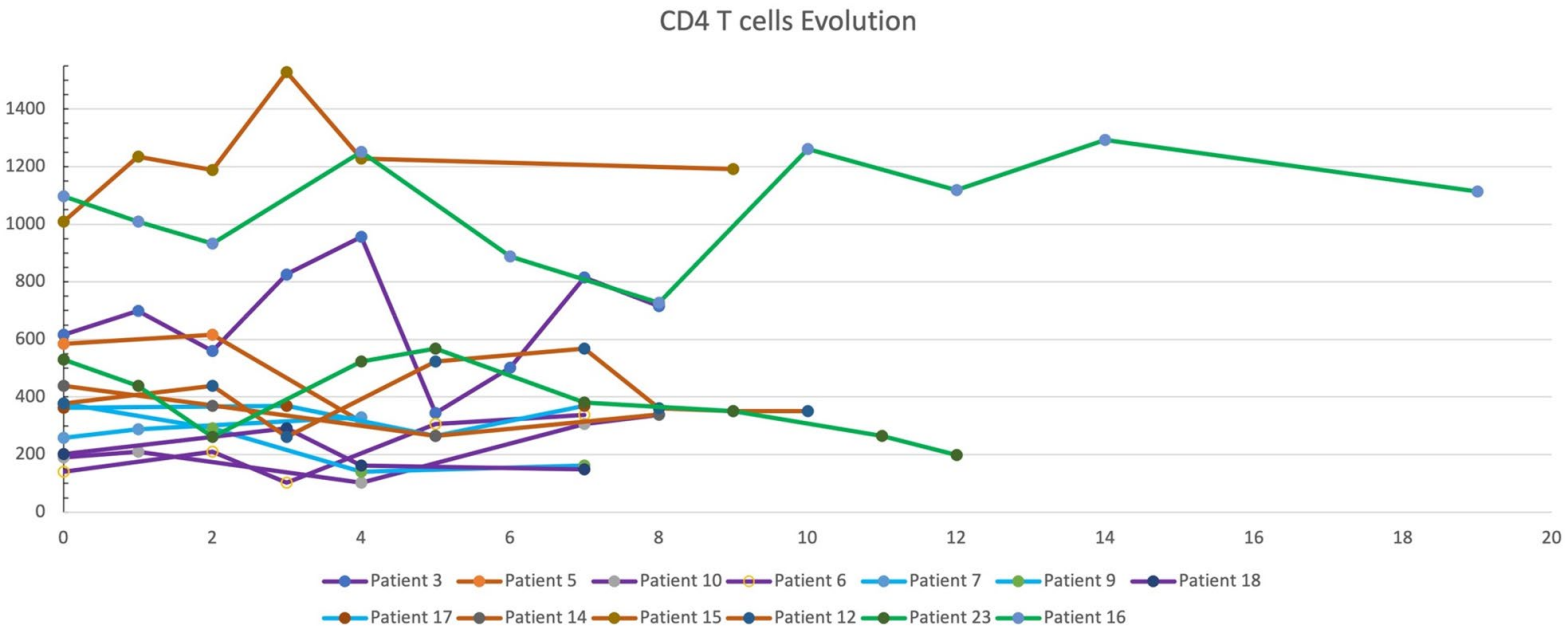
CD4 T cells evolution as a function time under Anti PD-1 therapy

From May 8, 2018, to December 10, 2021, 314 PLWH with cancer have been presented. Thirty-six patients were proposed for Anti-PD-1 therapy, 2 selected other anti-PD-1 medicine, 2 with a low CD4^+^ T-cell count (< 100 cells/µL), and 8 were in other clinical trials, were excluded. A total of 24 patients were included in this study. (Table 1) The median age (range) was 59 (41–75) years. 21 (87.5%) participants were men and 3 (12%) were women. They are all Chinese, twenty-two of them Han, 1 Mongolian, and 1 Uygur. The median CD4^+^ T-cell count was 528/µL (range, 125–1309 cells/µL). Twenty-three (96%) received ART before the Anti-PD-1 therapy. No change in HIV viral load was changed during follow-up. CD4^+^ T-cell counts were monitored during treatment in 13 patients. Figure 1 showed the evolution of CD4^+^ T-cell counts in 13 patients who had value under camrelizumab. There is no significant change in CD4^+^ T-cell count between the baseline and the time of the last treatment (p = 0.786). Overall, there was no obvious change.

**Table 1:** Basic HIV Medical history and clinical characteristics

	Patients (N = 24) (% or median)
Median age	59 (41–75)
Gender	
Male	21 (88%)
Female	3 (12%)
CDC	
A	6 (25.0%)
B	5 (21%)
C	3 (12%)
Unknown	10 (42%)
ECOG performance status	
0	15 (62%)
1	9 (37%)
Baseline CD4^+^ count	528.3/uL (125–1309)
Baseline CD4^+^/CD8^+^ ratio	1.26 (0.22–4.12)
ART	
With	23 (96%)
Without	1 (4%)
ART regimen	
PI-based regimen	15 (62%)
NNRTI-based regimen	8 (32%)
INSTI-based regimen	1 (4%)
Duration of ART (year)	2.94 (0–9)
Baseline HIV viral load	
Undetectable	23 (96%)
>40 copies/mL	1 (4%)
Baseline laboratory test (before ICPs)	
WBC	8.16 (2.68029.9)
HGB	123.91 (66–160)
PLT	246.67 (68–539)
ALT	21.88 (7–87)
AST	25.88 (13–74)
TBIL	14.80 (4.5–42.3)
Serum creatine (umol/L)	112.87 (47–933)
eGFR	88.217 (4.7-116.3)
Combined with chemotherapy	
Yes	6 (25%)
No	18 (75%)
Previous therapy	
Radiotherapy	2 (8%)
Chemotherapy	6 (25%)
Surgery	10 (42%)
None	6 (25%)

*ECOG* eastern cooperative oncology group,* ART* active antiretroviral therapy,* PI* protease inhibitor;* NNRTI* non-nucleoside reverse transcriptase inhibitors,* INSTI* integrase strand transfer inhibitor,* eGFR* estimated glomerular filtration rate

### Safety outcomes

Safety was noted over the course of 207 cycles in 24 participants. The median number of cycles was 8 (range, 4–26). At the time of analyses, Five participants continued to receive Anti-PD-1 therapy. Treatment-emergent AE s at least possibly attributed to Camrelizumab that occurred in participants are presented in Table 2. Most AEs were grade 1 or 2 (n = 22), with 2 (8%) being grade 3. Reactive cutaneous capillary endothelial (RCCEP) was the most frequent AE and Grade 3 of RCCEP occurred in 1 patient with non-Hodgkin lymphoma. And 1 patient’s grade 3 of lymphocyte count decreased. No toxic deaths have been recorded.

**Table 2:** Adverse events

	No,(%) of patients	≥Grade 3
	Any grade	
RCCEP	20 (83%)	1(4%)
Hypothyroidism	1 (4%)	0
Asthenia	2 (8%)	0
Anaemia	2 (8%)	0
WBC count decreased	3 (13%)	0
Decreased appetite	4 (17%)	0
Diarrhoea	1 (4%)	0
Neutrophil count decreased	2 (8%)	0
Lymphocyte count decreased	1 (4%)	1(4%)
Nausea	5 (21%)	0
Vomiting	3 (13%)	0
Hyponatraemia	4 (17%)	0
Lung infection	1 (4%)	0
Febrile neutropenia	1 (4%)	0
Death	0	0

Data are n (%). *RCCEP* reactive cutaneous capillary endothelial

### Tumor type and response

**Fig. 2:**
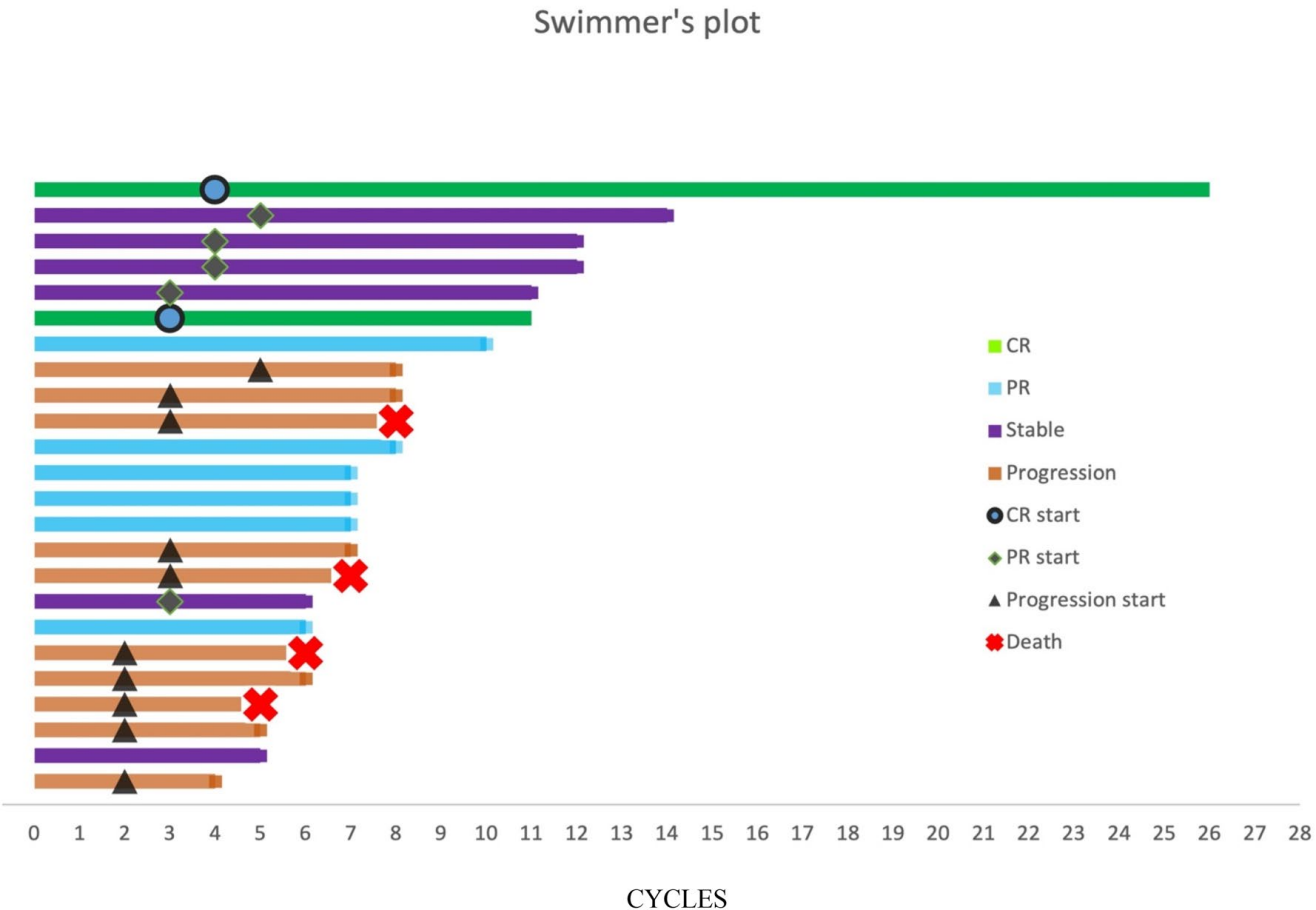
Swimmer’s plot : Duration of tumor response in cycles (Camrelizumab)

**Fig. 3:**
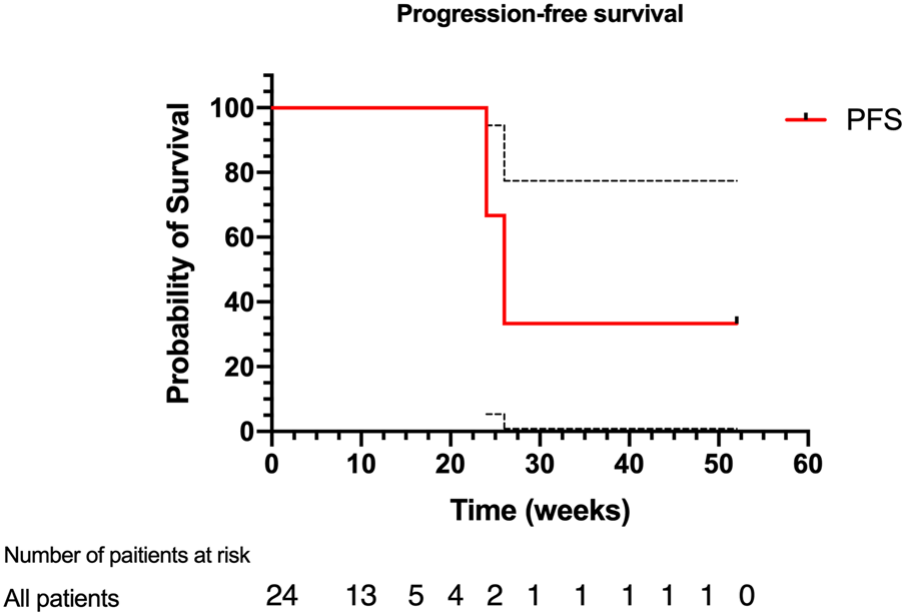
Kaplan-Meier estimates of progression-free survival

The tumor types and response were presented in Table 3. The swimmer plot of duration response was shown in Fig. 2. Eighteen (75%) patients were treated with monotherapy. The most tumor type in this study was bladder cancer (6%). Unfortunately, nine had progressed and a total of 4 patients in the study died at the time of analysis. Defined clinical benefit (CR and PR) was noted in 2 (8%) with bladder cancer and 6 (25%) participants, respectively. The median duration of response in these 8 responding patients was 9 weeks. Seven patients had stable tumor status, and others progressed (Fig. 2). A total of 7 patients underwent genetic testing, where PD-L1 expression was > 1% in 4 patients and < 1% in the remaining 3 patients. Six patients received the combined treatment with camrelizumab and chemotherapy. The progression-free survival (PFS) for all cohorts is shown in Fig. 3.

**Table 3:** Cancer Characteristics; treatment response assessment

Patient	Tumor type	PD-1 cycle	Response	Survival (yes/no)	TMB (mutes/Mb)	MSI	PD-L1 status (CPS)	Combined chemotherapy (yes/no)
#1	Seminoma	4	Progression	Yes	N/A	N/A	N/A	No
#2	Gastric cardia cancer	5	Stable	Yes	N/A	N/A	N/A	No
#3	Renal carcinoma	8	Progression	Yes	2.74	MSS	< 1	No
#4	Rectal carcinoma	7	Progression	No	N/A	N/A	N/A	No
#5	Non-Hodgkin lymphoma	8	PR	Yes	8.21	MSS	20	Yes
#6	Anal cancer	6	Progression	Yes	N/A	N/A	N/A	No
#7	Non-Hodgkin lymphoma	7	PR	Yes	N/A	N/A	N/A	Yes
#8	Cervical cancer	6	Stable	Yes	N/A	N/A	N/A	No
#9	Hepatocellular carcinoma	7	PR	Yes	N/A	N/A	N/A	No
#10	Renal carcinoma	8	Progression	No	N/A	N/A	N/A	No
#11	Renal pelvic cancer	6	Progression	No	22.80	MSS	< 1	No
#12	Gastric cancer	12	Stable	Yes	N/A	N/A	N/A	Yes
#13	Renal carcinoma	14	Stable	Yes	N/A	N/A	N/A	No
#14	Penile carcinoma	11	Stable	Yes	N/A	N/A	N/A	No
#15	Bladder cancer	12	Stable	Yes	7.30	MSS	< 1	No
#16	Bladder cancer	26	CR	Yes	4.56	MSS	10	Yes
#17	Bile duct cancer	10	PR	Yes	N/A	N/A	N/A	No
#18	Bladder cancer	7	Progression	Yes	N/A	N/A	N/A	No
#19	Bladder cancer	5	Progression	No	N/A	N/A	N/A	No
#20	Ureteral cancer	8	Progression	Yes	20.98	MSS	2	No
#21	Hepatocellular carcinoma	7	PR	Yes	17.33	MSS	80	No
#22	Bladder cancer	6	Stable	Yes	N/A	N/A	N/A	Yes
#23	Bladder cancer	11	CR	Yes	N/A	N/A	N/A	Yes
#24	Renal cancer	6	PR	Yes	N/A	N/A	N/A	No

## Discussion

In the last few years, cancer immunotherapy has significantly advanced. Immune checkpoint proteins, like PD-1, have taken an active role in mediating T-cell exhaustion both in cancer and chronic infections. Upon interaction with their ligands, for example, PD-L1, a negative signaling cascade is activated within the T cell that blocks T cell receptor-mediated activation. Consequently, the T cell loses its effector function, and thus, its ability to destroy cancer cells or provide cytokines for proper immune homeostasis. Once the interaction between the checkpoint protein and its ligand is interrupted, for example, by a blocking antibody, T-cells can regain effector functions and participate again in cancer surveillance [14]. Anti-PD1 therapy has been approved for a variety of cancers that increased incidence in PLWH, including squamous cell skin cancer [15], cervical cancer [16], lung cancer [17, 18], Hodgkin lymphoma [19, 20] and hepatocellular carcinoma [21].

In this study, CD4 T-cell count levels were found to fluctuate, slightly increasing or decreasing, and at the last therapy cycle, there were 3 patients with levels that were lower than the baseline level. Our results showed that camrelizumab had no detrimental effect on CD4 T-cell counts. The previous study [6]. found CD4 T-cell had a median increase of 19 cells/µL in HIV patients treated with pembrolizumab. Spano’s study of 23 HIV patients treated with anti-PD1 therapy reported a slight decrease in CD4 T-cell count [22]. Potential concerns have included the administration of increased expression of PD-1 in HIV infection, which is inversely correlated with CD4 T-cell count. In a case report of an HIV-positive patient with advanced lung cancer, nivolumab injections increased HIV-specific CD8 T-cells and a drastic and transient diminution of the HIV reservoir [23]. However, our retrospective study did not allow us to assess the impact on the HIV reservoir or HIV-specific CD8^+^ T-cell.

The most common AE was reactive capillary endothelial proliferation (RCEP) (238 [79.9%]. vs. 32 [10.8%].) in the previous study, this adverse event mainly occurred at grade 1 or 2 [24]. Most patients with RCEP did not require special treatment, and it spontaneously regressed after discontinuing camrelizumab. RCEP is considered an immune response of capillary endothelial cells. Previous studies have been reported that its occurrence is positively associated with tumor response [25]. The proportion of grade 3 and 4 AEs was similar to that previously reported in general patients receiving anti-PD-1 therapy [17, 18, 24]. The outcomes in this research are consistent with previous ones. However, the safety of this therapy in PLWH has not been previously explored prospectively. This study confirms the primary safety of camrelizumab in PLWH with cancers.

Data on the tolerance and efficacy of Anti-PD-1 in PLWH are scarce. To the best of our knowledge, this is the first study that evaluated camrelizumab in PLWH with advanced cancers. Previous studies assessed the safety of pembrolizumab in PLWH and advanced cancers, and tumor regression in participants with a range of tumor types confirmed the activity of anti-PD-1 therapy in PLWH [6]. Spano et al. [22]. reported the efficacy and tolerance of nivolumab or pembrolizumab in PLWH with cancers. The observed clinical benefit rate in this study was 33.3% (PR + CR), which was very impressive. Immune checkpoint inhibitors provide an important treatment option for PLWH with cancer [7, 26]. Unfortunately, anti-PD1-related data on the tolerance and efficacy of ICIs in PLWH is scarce, as these patients are usually excluded from clinical trials [24, 27]. Expanding clinical trial eligibility to include patients with HIV is justified in most cases and may accelerate the development of effective therapies in this area of unmet clinical need. Currently, there are several ongoing clinical trials to evaluate immune checkpoint inhibitors in PLWH, like DURVAST, NCT03094286, and NCT02869789. Results of these ongoing clinical trials are awaited to determine and hopefully address the safety and efficacy of anti-PD-1 therapy in PLWH.

### Study limitation

The retrospective study design and sample size did not allow for a formal comparison of rates of specific AEs in the general population. In addition, this study was not randomized. Although this study demonstrated camrelizumab had a clinical benefit in PLWH with several cancers, this study did not have enough participants with a tumor to evaluate the tumor response accurately.

## Conclusion

Data from the present study strongly support the use of camrelizumab (monoclonal antibodies targeting the PD-1 pathway) in this population, as it appears to be a feasible approach with no deleterious effects on PLWH and tolerability and acceptable efficacy. In addition, these findings further support the inclusion of PLWH with cancer in clinical trials evaluating the safety and efficacy of ICIs on cancer.

## Data Availability

All generated and available data for this paper has been included.
